# Genetic Identification of Burned Human Remains: A Systematic Review

**DOI:** 10.3390/genes17080881

**Published:** 2026-07-28

**Authors:** Francesco Sessa, Martina Francaviglia, Emina Dervišević, Pietro Zuccarello, Mario Chisari, Serena Matera, Grazia Giulia Panté, Monica Salerno, Massimiliano Esposito

**Affiliations:** 1 Department of Psychology and Health Sciences, Faculty of Human Sciences, Education, and Sports, Pegaso University, 80143 Naples, Italy; pietro.zuccarello@unipegaso.it; 2 Department of Medical, Surgical and Advanced Technologies “G.F. Ingrassia”, University of Catania, 95121 Catania, Italy; martinafrancaviglia95@gmail.com (M.F.); graziagiuliapante@gmail.com (G.G.P.); monica.salerno@unict.it (M.S.); 3 Department of Forensic Medicine, Faculty of Medicine, University of Sarajevo, 71000 Sarajevo, Bosnia and Herzegovina; emina.dervisevic@mf.unsa.ba (E.D.); massimiliano.esposito@unikore.it (M.E.); 4 Faculty of Medicine and Surgery, “Kore” University of Enna, 94100 Enna, Italy; mario.chisari@unikore.it; 5 Department of Clinical and Experimental Medicine, Section of Occupational Medicine, University of Catania, 95123 Catania, Italy; serena.matera@unict.it

**Keywords:** forensic sciences, DNA analysis, degraded human remains, burned remains, autosomal STRs, Y-STRs, mitochondrial DNA, single-nucleotide polymorphisms (SNPs), massively parallel sequencing (MPS), rapid DNA technologies

## Abstract

**Background/Objectives**: DNA-based identification of degraded human remains represents a major challenge in forensic science, particularly in cases involving burned, fragmented, or commingled bodies. Advances in forensic genetics have expanded the analytical capabilities for such samples; however, the effectiveness of different approaches and their integration within Disaster Victim Identification (DVI) workflows remain heterogeneous. This systematic review aims to critically evaluate current evidence on DNA-based identification of degraded remains, focusing on methodological strategies, emerging genomic technologies, and DVI applications, while integrating laboratory evidence and operational forensic practice into a structured analytical framework. **Methods**: A systematic literature search was conducted in Scopus and Web of Science from database inception to 5 June 2026, following PRISMA 2020 guidelines. Eligible studies included original research addressing DNA analysis of degraded, thermally altered, or highly compromised human remains in forensic or DVI contexts. After a multistep screening process involving title/abstract and full-text evaluation, 37 studies were included. Data were extracted and organized into three thematic categories: (i) core DNA analysis, (ii) advanced molecular technologies, and (iii) DVI case applications. **Results**: The findings demonstrate that DNA recovery from degraded remains is influenced by thermal exposure, tissue type, and sampling strategy. Teeth and dense cortical bone consistently provide higher DNA yield. While autosomal STR profiling remains the primary analytical approach, its limitations in highly degraded samples are mitigated through the complementary use of mitochondrial DNA (mtDNA), Y-chromosome STRs (Y-STRs), and SNP markers, together with advanced sequencing technologies such as massively parallel sequencing (MPS). Emerging technologies, including rapid DNA systems and predictive models based on macroscopic indicators, significantly enhance efficiency and success rates. DVI studies report identification rates exceeding 90–95% when multidisciplinary and structured workflows are applied. The evidence further supports a flexible triage-based analytical strategy, in which marker selection is guided by tissue preservation and degradation level. **Conclusions**: DNA-based identification of degraded human remains has evolved into an adaptive, multi-level forensic process. Successful outcomes rely on the integration of optimized sampling, hierarchical genetic analysis, and coordinated DVI strategies. The findings support a triage-based framework that links tissue selection, degradation assessment, and analytical methodology to maximize identification success. Future developments should focus on predictive models, advanced genomic tools, and standardized workflows to further improve identification in challenging forensic scenarios.

## 1. Introduction

The identification of human remains represents a fundamental objective in forensic science, particularly in contexts involving criminal investigations, humanitarian operations, and mass fatality incidents. Establishing the identity of deceased individuals is essential not only for legal and judicial purposes but also for providing closure to families and enabling appropriate cultural and religious practices related to burial and mourning [1]. The importance of reliable identification becomes even more critical in scenarios where conventional methods such as visual recognition, fingerprint analysis, or dental comparison are no longer applicable due to severe alteration of the body.

Among the most challenging conditions encountered in forensic practice are those involving degraded, burned, fragmented, or commingled human remains, typically resulting from high-energy events such as aviation disasters, explosions, fires, natural disasters, or armed conflicts. Exposure to extreme temperatures can lead to extensive destruction of soft tissues, fragmentation of skeletal elements, and severe degradation of biological molecules, including DNA. Thermal insult, in particular, induces strand breakage, oxidative damage, and cross-linking, drastically reducing the quantity and quality of recoverable genetic material [2,3,4,5].

In such scenarios, forensic genetics has emerged as the most reliable and often the only viable approach for identification. DNA analysis allows the comparison of postmortem biological samples with antemortem reference material obtained either directly from the individual (e.g., personal items) or indirectly through biological relatives. The introduction of short tandem repeat (STR) analysis has revolutionized forensic identification due to its high discriminatory power, even in degraded samples [6,7,8,9,10,11]. However, the success of STR-based profiling is strongly influenced by DNA preservation, and in highly compromised remains, partial profiles, allelic dropout, or complete profiling failure are frequently observed.

To overcome these limitations, additional genetic markers and analytical approaches have been progressively integrated into forensic workflows, including Y-chromosome STRs (Y-STRs), mitochondrial DNA (mtDNA), and single-nucleotide polymorphisms (SNPs). These markers can be investigated using different technological platforms, including capillary electrophoresis (CE), Sanger sequencing, and, more recently, massively parallel sequencing (MPS) technologies. Y-STR analysis is particularly useful for paternal lineage tracing in male individuals, while mtDNA, due to its high copy number and resistance to degradation, provides valuable information for maternal lineage identification when nuclear DNA is insufficient [12,13,14,15,16,17,18]. Furthermore, MPS, also referred to as next-generation sequencing (NGS), has expanded the analytical capabilities of forensic genetics by enabling the analysis of multiple marker systems, including STRs, mtDNA, and SNPs, from highly degraded samples [19,20,21,22,23].

In parallel with technological advancements, the field of Disaster Victim Identification (DVI) has evolved into a highly structured, multidisciplinary process. International guidelines, particularly those developed by INTERPOL, emphasize a systematic approach integrating forensic pathology, anthropology, odontology, and genetics to ensure accurate and reliable identification. Similarly, the International Committee of the Red Cross (ICRC) has promoted the application of forensic science for humanitarian purposes, highlighting the importance of standardized procedures for the management of the dead, identification of missing persons, protection of human dignity, and support for affected families in both disaster and conflict settings [24,25,26]. Within this framework, DNA analysis plays a central role, consistent with both INTERPOL DVI standards and ICRC recommendations for the identification of unknown deceased persons and missing individuals in humanitarian contexts [26,27,28].

Recent years have also witnessed a paradigm shift in operational forensic practice with the introduction of rapid DNA technologies, which enable the generation of genetic profiles within a few hours. These systems have proven particularly valuable in time-sensitive scenarios such as mass casualty events, where rapid identification is crucial for both humanitarian and logistical reasons [29]. However, despite these advantages, rapid DNA platforms may exhibit lower analytical sensitivity than conventional (CE)-based workflows and, particularly in highly degraded or low-template samples, may not achieve the performance currently attainable with MPS technologies. Significant challenges therefore remain regarding sample selection, DNA degradation, contamination risks, interpretation of complex or mixed genetic profiles, and broader implementation in forensic practice.

Despite significant advances in forensic genetics, the literature remains fragmented, with many studies focusing either on experimental degradation models or isolated case reports. Moreover, the rapid evolution of molecular techniques, including MPS and rapid DNA platforms, has not been systematically integrated with operational DVI practices. This gap highlights the need for a comprehensive and updated synthesis that connects biological evidence, analytical methodologies, and real-world forensic workflows.

To our knowledge, no previous systematic review has specifically integrated evidence on DNA recovery from burned human remains with emerging genomic technologies and operational DVI workflows. Most available publications focus either on methodological aspects of DNA degradation or on isolated forensic case reports. The present review therefore seeks not only to summarize the literature but also to bridge experimental findings, technological innovations, and practical forensic applications. By integrating these dimensions, we propose a structured framework for selecting analytical strategies according to the degree of thermal damage and the operational context of the investigation.

The aim of this systematic review is to critically evaluate and synthesize current evidence on DNA-based identification of burned human remains. Specifically, this review seeks to (i) assess the effectiveness of different genetic approaches, including autosomal STRs, lineage markers, and emerging technologies; (ii) identify key methodological and operational challenges associated with burned samples; and (iii) analyze their application and performance in forensic scenarios, highlighting recent advances and future perspectives in forensic genetics.

## 2. Materials and Methods

### 2.1. Study Design and Search Strategy

This study was designed as a systematic review aimed at evaluating the current state of forensic genetic approaches for human identification from burned and burnt human remains, with particular focus on DNA-based methodologies including STR profiling, SNP genotyping, and MPS.

The review was conducted in accordance with the Preferred Reporting Items for Systematic Reviews and Meta-Analyses (PRISMA) guidelines [30,31].

The search strategy was designed to capture studies addressing genetic identification techniques applied to thermally altered human remains.

The following keywords were used:

- “Genetic investigation”;
- “DNA analysis”;
- “STRs”;
- “Profiling”;
- “DNA identification”;
- “SNP genotyping”;
- “NGS” or “Next-generation sequencing”.

Each keyword was systematically combined using the Boolean operator AND with the following terms:

- “Burned remains”;
- “Burnt human remains”.

Search queries were applied to the fields “title, abstract and Keywords” in Scopus and equivalent fields in WoS. The search strategy employed broad forensic genetics and burned-remains keywords to maximize retrieval of studies relevant to DNA-based identification. Articles addressing specific analytical approaches (e.g., mtDNA analysis) or particular forensic scenarios (e.g., DVI investigations and aviation disasters) were subsequently identified during the screening and full-text review phases when they fulfilled the predefined eligibility criteria. No restrictions on publication date were applied until 5 June 2026. Only articles published in English were considered.

### 2.2. Eligibility Criteria

Studies were deemed eligible for inclusion if they met specific criteria related to study design, subject matter, and methodological relevance. Only original peer-reviewed research articles were considered, ensuring the inclusion of primary data and experimentally grounded findings. Eligible studies were required to focus on human remains, or on materials and methodologies directly applicable to forensic human identification.

A central inclusion requirement was the application of DNA-based analytical approaches. This encompassed a broad range of genetic methodologies, including autosomal STR profiling, Y-STR profiling, mtDNA sequencing, SNP genotyping, DNA methylation analysis, and applications based on MPS technologies. Studies employing these methods were included provided they contributed to the identification or characterization of degraded biological material.

Furthermore, studies were required to address thermally altered, burned, charred, incinerated, or otherwise highly degraded human remains, or to present methodologies explicitly applicable to such conditions. The forensic context was also a key criterion, and eligible studies included those conducted in forensic casework or DVI settings, including experimental studies, methodological investigations, and case reports.

Exclusion criteria were defined to ensure methodological consistency and relevance. Non-original publications, including review articles, book chapters, books, conference papers, and brief notes, were excluded. Studies that did not involve DNA analysis, such as those focused solely on anthropological, odontological, or imaging techniques, were also removed from consideration.

Additionally, studies unrelated to forensic human identification were excluded, including those focused on environmental or ecological applications, plant or animal research, clinical or biomedical investigations on burn victims, and microbiological or pathogen-centered studies.

Studies lacking clear relevance to degraded or thermally altered remains were excluded during the screening process. Finally, at the full-text stage, articles were excluded only when DNA analysis was not performed or did not constitute a central component of the investigation. Studies reporting unsuccessful DNA recovery, partial genetic profiles, amplification failure, or other negative analytical outcomes were retained when they provided information relevant to forensic identification, DNA degradation, or methodological performance.

### 2.3. Study Selection Process

The selection process was performed independently by two reviewers, and discrepancies were resolved through discussion. A total of 202 records were initially identified. After removal of 87 duplicates, primarily due to overlap between databases and repeated indexing of the same articles with minor metadata variations, 115 unique records were retained.

Following duplicate removal, 23 records were further removed, in order to prioritize the original articles.

92 records underwent a two-step screening process:

- Title and abstract screening to identify potentially relevant studies.
- Full-text assessment to confirm eligibility based on inclusion and exclusion criteria.

After this screening, 54 articles were excluded for the following reasons: lack of forensic genetic relevance (n = 19), absence of DNA-based analysis (n = 11), non-human or non-forensic context (n = 9), and unrelated topics such as environmental, clinical, or ecological research (n = 15). This screening step resulted in 38 articles being considered eligible for full-text assessment.

Full-text versions of these articles were retrieved and carefully evaluated for methodological relevance, the applicability of DNA-based identification, and the focus on degraded or thermally altered human remains. At this stage, 1 study was excluded because DNA analysis was not a central methodological component of the investigation.

**Figure 1:**
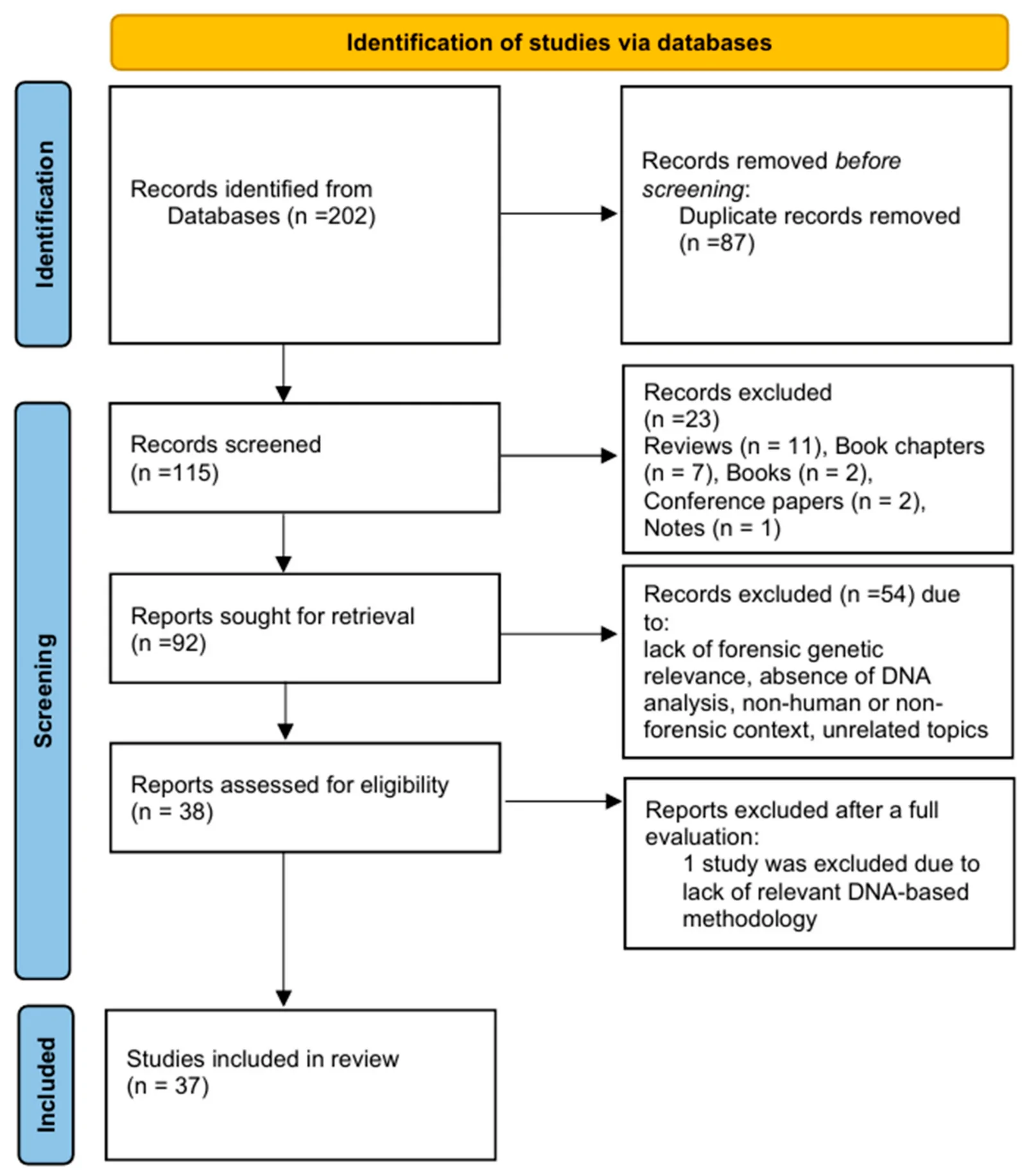
The study selection followed a multistep screening process in accordance with PRISMA guidelines.

Ultimately, a total of 37 studies met all inclusion criteria and were included in the qualitative synthesis. A PRISMA flow diagram summarizing the study selection process, including identification, screening, eligibility, and inclusion phases, is presented in Figure 1.

### 2.4. Data Extraction and Synthesis

Data extraction was performed for all included studies using a standardized and systematic approach to ensure consistency and comparability across heterogeneous study designs. For each article, detailed information was collected regarding the authorship and year of publication, as well as the type of study, which was categorized as experimental research, case report, or methodological investigation.

Particular attention was devoted to the characterization of the biological material analyzed, including the type of tissue examined, such as bone, teeth, or soft tissue, and the degree of thermal alteration or degradation reported. Furthermore, the specific DNA-based methodologies applied in each study were systematically recorded. These included genetic marker systems and analytical approaches such as autosomal STR profiling, Y-STR profiling, mtDNA sequencing, SNP genotyping, DNA methylation analysis, and MPS-based workflows.

Finally, the main outcomes of each study were extracted, with a focus on parameters relevant to forensic identification, including DNA recovery efficiency, genetic profiling success, and any methodological advancements or innovations proposed by the authors.

### 2.5. Thematic Classification

To facilitate a structured and coherent synthesis of the evidence, the included studies were subsequently organized into three main thematic categories based on their primary focus.

The first category comprised studies investigating the fundamental aspects of DNA degradation and recovery in burned or highly degraded remains. These studies primarily addressed the mechanisms of DNA damage, the impact of thermal exposure, and the performance of STR-based profiling in compromised biological samples.

The second category included studies focused on advanced genetic technologies, encompassing the application and evaluation of cutting-edge methodologies such as NGS, SNP-based analyses, whole mitochondrial genome sequencing, epigenetic approaches, and rapid DNA platforms. These studies provided insight into the technological evolution of forensic genetics and its capacity to overcome the limitations associated with degraded DNA.

The third category comprised studies related to DVI and real-case forensic applications. This group included case reports and investigations conducted in mass fatality scenarios, providing valuable information on the practical implementation of DNA-based identification strategies in complex operational settings.

### 2.6. Quality Considerations

Due to the heterogeneity of the included studies in terms of design, objectives, and methodological approaches, the application of a formal risk-of-bias assessment tool was not considered appropriate. Nevertheless, all studies were critically evaluated to ensure scientific rigor and relevance.

Each study was assessed based on methodological transparency, including the clarity and completeness of the analytical procedures described. Reproducibility was also considered, with emphasis on whether the reported methods could be reliably replicated in forensic practice. Additionally, the forensic applicability of the findings was evaluated, focusing on their relevance to real-world identification scenarios. Finally, the clarity and robustness of the DNA analytical procedures were examined to ensure that the reported results were supported by sound and well-described methodologies. Both successful and unsuccessful DNA analytical outcomes were considered informative for the purposes of this review, as they contribute to understanding the limitations and performance of forensic genetic approaches in degraded remains.

### 2.7. Study Quality Assessment

To provide a structured evaluation of the included studies, a qualitative scoring system was applied based on five criteria: (i) robustness of the DNA analytical method, (ii) sample size, (iii) control of degradation conditions, (iv) reproducibility of methods, and (v) applicability to DVI contexts. Each criterion was scored on a scale from 1 (low) to 5 (high).

The scoring system represents a qualitative assessment and should not be interpreted as a ranking of study quality. Studies were not excluded based on quality; however, this framework was used to support the interpretation of the results and to identify methodological strengths and limitations across the literature.

## 3. Results

A total of 37 studies were included in the qualitative synthesis, spanning a publication period from 1995 to 2026. The included studies encompassed a wide range of research designs, including experimental investigations, methodological studies, and real-case applications in forensic and DVI contexts (Table 1).

Across the included studies, bone and teeth represented the most frequently investigated sample types, reflecting their superior resistance to thermal and environmental degradation. Soft tissues, blood, and mixed biological specimens were examined less frequently and were generally available only in partially preserved remains. Success criteria varied among studies and included complete STR profiles, partial genetic profiles sufficient for kinship analysis, successful mtDNA sequencing, SNP recovery, or final positive identification within a forensic or DVI investigation. This heterogeneity precluded formal quantitative comparison but consistently demonstrated higher success rates when sampling focused on protected biological substrates such as teeth and dense cortical bone.

The analyzed literature reflects the progressive evolution of forensic genetics from early polymerase chain reaction (PCR)-based approaches to advanced genomic technologies, including NGS, rapid DNA systems, and epigenetic applications.

The studies were categorized into three main thematic areas:

(i) Core DNA recovery and analysis in burned and degraded remains;
(ii) Advanced genetic and analytical technologies;
(iii) Real-case applications in DVI scenarios.

Overall, the analyzed studies demonstrate that DNA-based identification of degraded human remains has evolved from basic STR analysis to a highly integrated, multi-level forensic approach, combining advanced molecular techniques, optimized sampling strategies, and large-scale operational frameworks to achieve high identification success rates even under extreme conditions.

### 3.1. Qualitative Assessment of Study Quality

To support the interpretation of the included studies, a qualitative scoring framework was applied based on five criteria: robustness of the DNA analytical method, sample size, control of degradation conditions, reproducibility of methods, and applicability to DVI contexts. A detailed qualitative assessment of the included studies is provided in Supplementary Table S1.

Experimental studies investigating DNA degradation and recovery generally showed high scores in terms of reproducibility and control of degradation conditions (score 4–5), reflecting their controlled laboratory settings. However, these studies were typically characterized by limited sample sizes and reduced applicability to real DVI scenarios (score 2–3).

Studies focused on advanced genetic technologies, including MPS and predictive models, demonstrated the highest methodological robustness (score up to 5) and good balance between reproducibility and applicability, although their implementation in routine forensic practice remains partially limited by cost and complexity.

Conversely, DVI and real-case studies achieved the highest scores in terms of applicability (score 5) and sample size but showed greater variability in methodological control and reproducibility due to the inherent complexity and unpredictability of real-world forensic scenarios.

Overall, this assessment highlights a complementary relationship between experimental, technological, and operational studies, with each category contributing distinct strengths to the field of forensic genetic identification.

### 3.2. Core DNA Analysis in Burned and Degraded Remains

Early studies demonstrated the feasibility of DNA recovery from thermally altered tissues. Sweet and Sweet (1995) reported successful DNA extraction from dental pulp in incinerated remains, highlighting the protective role of dental tissues under extreme thermal conditions [32]. Similarly, Strom and Rechitsky (1998) applied nested PCR to identify charred remains, emphasizing the importance of highly sensitive amplification techniques [33].

Subsequent investigations confirmed that skeletal tissues remain a key substrate for DNA analysis. Staiti et al. (2004) demonstrated successful STR profiling from carbonized bone samples [38], while Kapińska and Szczerkowska (2004) reported that phenol–chloroform extraction methods yield superior results in degraded bone specimens [39].

However, DNA recovery is strongly influenced by thermal exposure. von Wurmb-Schwark et al. (2004) showed that DNA profiling from fully cremated remains (>1000 °C) is often unreliable due to extreme degradation and contamination [36]. This finding was later reinforced by Schwark et al. (2011), who systematically classified burned bones and demonstrated that STR success decreases progressively from partially burned to calcined remains [42].

Experimental studies further clarified degradation dynamics. Garriga et al. (2016) observed a sharp decline in STR amplification above 300 °C [45], while Lozano-Peral et al. (2021) confirmed preferential loss of longer DNA fragments and improved performance of mini-STR loci [50].

More recent studies emphasized the role of macroscopic indicators. Thibodeau-Cadieux et al. (2025) demonstrated that bone color and porosity correlate directly with DNA preservation [5], supporting earlier findings linking tissue condition to DNA yield.

Recent evidence suggests that fibrocartilage from human intervertebral disks may represent an additional valuable source of DNA in severely charred bodies. Tomsia et al. obtained complete autosomal and Y-STR profiles from all examined cases and successfully identified victims through kinship analysis, highlighting the potential of intervertebral disk fibrocartilage as a rapid and cost-effective alternative when conventional skeletal samples are unavailable or heavily damaged [68].

Collectively, these studies indicate that DNA recovery success is determined not only by the degree of thermal exposure but also by tissue-specific protective mechanisms. Across the reviewed studies, teeth and dense cortical bone consistently outperformed other substrates, whereas heavily calcined or porous skeletal elements showed markedly reduced profiling success. These findings suggest that pre-analytical tissue selection may be as important as downstream analytical methodology in determining identification outcomes.

### 3.3. Advanced Genetic and Analytical Technologies

Advances in forensic genetics have significantly improved identification success in degraded samples. MtDNA sequencing has been widely used as a complementary analytical approach when nuclear DNA is insufficient. Cerri et al. (2004) successfully applied mtDNA sequencing to identify burned remains in cases where STR results were inconclusive [37].

Similarly, Oh and Park (2022) demonstrated that mtDNA analysis remains feasible even in cremated remains from historical cases, highlighting its resilience under extreme thermal conditions [55].

Technological improvements in STR analysis have also enhanced performance. Elwick et al. (2018) showed that modern STR kits exhibit improved sensitivity and inhibitor tolerance [46], while Zgonjanin et al. (2019) reported near-complete profiling success using updated multiplex systems [48].

Innovative extraction methods have further contributed to improved DNA recovery. McKinnon and Higgins (2021) demonstrated that retaining EDTA supernatant significantly increases DNA yield from burned bone [51], and Varrathyarom et al. (2022) introduced bead-beating homogenization to improve extraction efficiency [53]. Similarly, Uzair et al. demonstrated that total demineralization and organic extraction methods outperform Chelex-based protocols for DNA recovery from burned human bones, emphasizing the importance of inhibitor removal and extraction strategy in successful STR profiling [69].

A major technological breakthrough is represented by ancient DNA (aDNA) methodologies. Emery et al. (2020) showed that aDNA protocols significantly improve recovery of ultrashort fragments from highly burned remains [49]. These analyses were subsequently enhanced through the adoption of MPS technologies. The successful recovery of SNP markers from highly degraded and thermally altered remains has also opened up the possibility of future applications extending beyond conventional identification, including forensic DNA phenotyping and other intelligence-led investigative approaches. Emery et al. (2022) reconstructed complete mitochondrial genomes from thermally altered remains [54]; subsequently, the same authors demonstrated successful SNP recovery from highly burned skeletal material [60].

Recent studies have introduced predictive and quantitative approaches. Rahmat et al. (2023) developed models to predict DNA recoverability based on thermal exposure [57], while Macias et al. (2024) established a colorimetric framework to estimate DNA quantity and STR success in burned bone [59].

Rapid DNA technologies represent a further significant advancement. Snedeker et al. (2023) optimized automated extraction systems for degraded samples [58], and Weinthal et al. (2025) demonstrated the use of rapid DNA platforms capable of generating profiles within hours [66].

Finally, novel applications such as DNA methylation have expanded forensic capabilities beyond identification. Grignani et al. (2025) successfully applied epigenetic markers for age estimation in burned remains [62].

Collectively, these findings indicate that advanced genetic technologies significantly extend the analytical limits of forensic identification, with MPS and SNP-based approaches enabling the recovery of genetic information in cases where conventional STR analysis fails. Although these technologies offer substantial advantages for degraded samples, their broader implementation in routine forensic practice remains dependent on laboratory infrastructure, validation requirements, bioinformatic resources, personnel training, and integration into existing workflows.

### 3.4. DVI and Real-Case Applications

A substantial portion of the included studies addressed real-case scenarios in DVI contexts. Early case reports demonstrated the feasibility of DNA identification in burned remains. Wickenheiser et al. (1999) successfully identified severely burned remains using STR-based paternity analysis [34], while Sivolap et al. (2001) reported identification from heavily altered bone fragments [35].

More complex DVI scenarios highlight the importance of standardized approaches. Ricci et al. (2015) described the identification of victims in a factory fire using STR and Y-STR analysis within an INTERPOL framework [43], while Zgonjanin et al. (2015) demonstrated the recovery of full profiles from burned skeletal remains [44]. Similarly, Bugoye et al. reported successful DNA-based identification of all twelve victims of a dormitory fire in Tanzania using autosomal STR analysis and kinship comparisons with relatives, highlighting the importance of multidisciplinary coordination and family reference sampling in DVI operations [70].

Recent case series have emphasized operational strategies. Gianfreda et al. (2025) reported successful identification of highly carbonized victims through optimized tissue selection [65], highlighting the importance of sampling strategies.

A landmark example was the 1998 Taoyuan Airbus crash in Taiwan, where DNA analysis of 685 fragmented remains enabled the reconstruction and identification of 202 victims. The study demonstrated the effectiveness of STR-based kinship analysis in large-scale aviation disasters involving severely fragmented and thermally damaged remains [71]. Large-scale disasters further demonstrate the effectiveness of modern forensic genetics. Ram et al. (2025) reported a 96% identification rate following a mass casualty event, using integrated genetic approaches [64], while Sarwar et al. (2026) achieved identification of 96 out of 97 victims in an aviation disaster using STR, Y-STR, and mtDNA combined with kinship analysis [67]. Aviation disasters have historically represented a major driving force in the development and validation of forensic DNA identification strategies [1,63]. Beyond the studies included in the present review, numerous aircraft-crash investigations since the 1990s have demonstrated the value of integrating genetic analyses with multidisciplinary DVI procedures, particularly when dealing with fragmented, burnt, and commingled remains [27]. These investigations have contributed substantially to the establishment of standardized identification protocols, the implementation of kinship-based identification strategies, and the progressive adoption of advanced molecular technologies in mass-fatality contexts [3].

Innovative approaches to reference samples have also been described. Chávez-Briones et al. (2023) successfully identified a victim using decades-old deciduous teeth [56], demonstrating the value of alternative biological references.

Notably, the reviewed DVI investigations demonstrate that analytical performance alone is insufficient to ensure successful victim identification. Instead, operational factors, including scene management, sample triage, reference collection, kinship databases, and interdisciplinary coordination, emerge as equally important determinants of identification success [72]. This finding highlights the transition from a laboratory-centered perspective toward an integrated DVI framework.

### 3.5. Comparative Effectiveness of Genetic Marker Systems and Analytical Technologies

A recurring source of confusion in forensic genetics is the distinction between genetic marker systems and analytical technologies. Genetic markers (e.g., autosomal STRs, Y-STRs, mtDNA, and SNPs) represent the biological targets examined, whereas analytical technologies (e.g., capillary electrophoresis and massively parallel sequencing) are the methodological platforms used to analyze them. These concepts are complementary rather than interchangeable and should be considered separately when evaluating identification performance.

Autosomal STR profiling remains the primary tool for forensic identification due to its high discriminatory power. However, its effectiveness is significantly compromised in highly degraded samples, where DNA fragmentation and inhibitor presence frequently result in partial or failed profiles [73].

MtDNA analysis demonstrates greater resilience to degradation due to its high copy number, allowing successful profiling in cases where nuclear DNA is severely compromised. Nevertheless, its lower discriminatory power and maternal inheritance limit its individualization capacity [74].

Y-chromosome STRs provide an additional lineage-based tool, particularly useful in male identification and kinship analysis, although their applicability is restricted to paternal lineage and they offer lower discrimination compared to autosomal STRs [75].

NGS and SNP-based approaches represent the most advanced methodologies, enabling the recovery of genetic information from highly degraded samples through the analysis of short DNA fragments. These techniques offer high sensitivity and expanded analytical potential and are increasingly applied to highly degraded forensic samples. While economic considerations remain relevant, recent evidence suggests that the primary challenges to broader implementation are often related to infrastructure requirements, laboratory validation, accreditation procedures, bioinformatic analysis, personnel training, data storage, and integration with existing forensic workflows rather than sequencing costs alone. Moreover, continuing technological advances have progressively reduced sequencing costs and improved operational efficiency [14,76,77].

Overall, the reviewed evidence suggests a progressive relationship between degradation level and analytical strategy. Autosomal STRs remain the preferred first-line approach when sufficient nuclear DNA is preserved. As degradation increases, mtDNA and Y-STRs provide complementary lineage information, while SNP-based MPS approaches offer the greatest analytical potential for highly degraded samples due to their ability to target shorter DNA fragments. Therefore, no single marker system can be considered universally optimal; rather, successful identification depends on matching the analytical approach to the biological condition of the remains.

## 4. Discussion

This systematic review provides a comprehensive and updated synthesis of the current evidence on DNA-based identification of degraded human remains, highlighting the progressive evolution of forensic genetics from early PCR-based methods to advanced genomic and rapid analytical approaches. The included studies collectively demonstrate that the success of identification in burned, fragmented, and commingled remains is governed by a complex interaction of biological, technical, and operational factors rather than DNA preservation alone. As shown in Supplementary Table S1, DVI studies achieved the highest scores for applicability and sample size, whereas experimental studies demonstrated greater reproducibility and control of degradation variables. Advanced genomic studies consistently showed the highest methodological robustness.

Beyond synthesizing the available literature, this review highlights the progressive convergence of laboratory research and operational forensic practice. A key contribution of the present work is the integration of evidence derived from controlled experimental studies, technological developments, and real DVI operations into a unified interpretative framework. This approach allowed for the identification of recurring factors associated with successful DNA recovery and facilitated the development of a hierarchical strategy that links sample condition, tissue selection, and analytical methodology. The importance of multidisciplinary and standardized identification procedures is also reflected in the humanitarian forensic framework promoted by the ICRC. In addition to supporting DNA-based identification, the ICRC emphasizes the integration of forensic science, information management, family engagement, and respectful handling of human remains as essential components of identification processes. These principles complement the operational DVI standards established by INTERPOL and reinforce the need for coordinated forensic strategies in mass-fatality incidents and humanitarian emergencies [26,28].

Early studies established the feasibility of DNA recovery from thermally altered tissues, particularly from protected substrates such as teeth and cortical bone, which have since been consistently recognized as the most reliable sources of genetic material in degraded remains [32,33].

Subsequent studies clarified that DNA degradation is strongly dependent on thermal exposure. von Wurmb-Schwark et al. (2004) demonstrated that DNA profiling from fully cremated remains (>1000 °C) is highly unreliable [36], while Schwark et al. (2011) systematically showed that STR success decreases progressively with increasing burn severity, with calcined bones yielding minimal or no nuclear DNA [42].

Experimental evidence further defined degradation thresholds. Garriga et al. (2016) reported a drastic decline in STR amplification above 300 °C [45], and Lozano-Peral et al. (2021) confirmed preferential degradation of longer DNA fragments, reinforcing the relevance of mini-STR approaches [50]. More recently, Thibodeau-Cadieux et al. (2025) demonstrated a direct correlation between macroscopic bone changes (color and porosity) and DNA preservation [5]. Comparative experimental work has also suggested that extraction efficiency in thermally altered bone and tooth samples can be improved through modified protocols combining silica-column purification and phenol–chloroform processing, supporting the importance of pre-analytical optimization in highly degraded specimens [78]. Recently, Haarkötter et al. reported superior overall DNA recovery using phenol–chloroform extraction, while silica-column approaches showed the highest extraction efficiency relative to sample input, emphasizing the importance of protocol selection in challenging forensic specimens [79].

Taken together, these findings demonstrate that DNA degradation follows a predictable gradient linked to thermal exposure and tissue characteristics. Importantly, they challenge the concept that DNA absence reflects analytical failure, suggesting instead that unsuccessful results often stem from suboptimal sampling or processing strategies.

While autosomal STR profiling remains the cornerstone of forensic identification, its limitations in highly degraded samples are well established. Several studies included in this review demonstrated that STR success is strongly influenced by DNA fragmentation and the presence of inhibitors [46], with allele dropout and partial profiles frequently observed.

To overcome these limitations, complementary genetic marker systems, including mtDNA, Y-STRs, and SNPs, have been increasingly employed using a variety of analytical platforms. Cerri et al. (2004) demonstrated the successful use of mtDNA in burned remains when STR analysis failed [37], while Oh and Park (2022) confirmed that mtDNA analysis remains feasible even in cremated historical remains, highlighting its resilience under extreme degradation conditions [55].

Advances in DNA extraction have also significantly improved outcomes. McKinnon and Higgins (2021) showed that retaining EDTA supernatant increases DNA yield from burned bone [51], and Varrathyarom et al. (2022) demonstrated enhanced efficiency using bead-beating homogenization [53]. Comparative studies have further shown that extraction methodology substantially influences profiling success, with total demineralization and organic extraction protocols generally providing superior DNA recovery from thermally altered bone compared with simpler extraction approaches [69].

A major paradigm shift is represented by the introduction of ancient DNA methodologies and MPS technologies, which enable the analysis of diverse marker systems from highly degraded samples. Emery et al. (2020) demonstrated that aDNA protocols significantly improve recovery of ultrashort DNA fragments in burned remains [49], while subsequent studies extended these findings to whole mitochondrial genome reconstruction [54] and SNP-based identification [60]. These approaches enable the recovery of genetic information even when conventional STR profiling fails completely.

Recent innovations also include predictive and quantitative approaches. Rahmat et al. (2023) proposed models to predict DNA recoverability from incinerated teeth based on thermal exposure [57], while Macias et al. (2024) demonstrated that colorimetric bone analysis can accurately predict DNA yield and STR success [59]. These studies mark a transition toward predictive forensic workflows, where analytical success can be estimated prior to laboratory processing.

Rapid DNA technologies represent a major operational advancement. Weinthal et al. (2025) demonstrated that rapid DNA systems can generate profiles within 2–3 h in mass casualty scenarios [66], significantly reducing turnaround times compared with conventional laboratory workflows. However, their application remains constrained by lower analytical sensitivity than standard CE-based methods and, particularly in highly degraded or low-template samples, by a reduced capability to recover genetic information compared with MPS-based approaches. Consequently, rapid DNA platforms are currently best suited as complementary tools for time-sensitive identification efforts rather than as replacements for comprehensive forensic genetic analyses [80,81,82].

An emerging application of SNP-based analysis that may become relevant in the context of severely burned and highly degraded remains is forensic DNA phenotyping (FDP). Unlike comparative DNA identification approaches, which rely on the availability of reference samples or relatives, FDP uses selected SNP markers to generate investigative information regarding externally visible characteristics, ancestry, and, in some cases, age-related features [83]. Recent advances in SNP recovery from highly degraded samples through MPS technologies suggest a potential future role for such approaches in cases where conventional comparative identification is unsuccessful or where suitable reference samples are unavailable [76,84,85]. In mass-fatality incidents or severely fragmented and commingled remains, SNP-based phenotyping may provide supplementary investigative leads that can help narrow the pool of possible identities while awaiting confirmatory identification through standard forensic methods. However, FDP should be regarded as a complementary investigative tool rather than a standalone identification technique. Furthermore, its application in heavily burned remains is insufficiently studied, and additional validation is required before routine implementation in DVI workflows. Nevertheless, the increasing ability to recover SNP data from degraded biological material supports continued investigation of FDP as a potential adjunct approach in challenging forensic identification scenarios.

A substantial proportion of the included studies addressed real-world DVI scenarios, demonstrating that high identification rates can be achieved even under extreme conditions. Wickenheiser et al. (1999) first showed successful identification of severely burned remains using STR-based kinship analysis [34], and similar findings were reported in subsequent DVI case studies [43,44].

More recent large-scale studies highlight the efficiency of integrated forensic workflows. Ram et al. (2025) reported identification rates of approximately 96% in a mass casualty event [64], while Sarwar et al. (2026) achieved identification of 96 out of 97 victims in an aviation disaster using combined STR, Y-STR, and mtDNA analysis with kinship-based approaches [67]. Although only a limited number of aviation-disaster studies met the inclusion criteria of the present review, aircraft-crash investigations, together with other large-scale mass-fatality incidents, have historically represented some of the most important applications of forensic DNA identification and have significantly influenced the evolution of modern DVI methodologies [19].

Importantly, these studies demonstrate that success is strongly dependent on operational strategy, including:

- Targeted sampling;
- Multi-sample analysis;
- Integration of multiple genetic systems;
- Probabilistic kinship analysis.

Additionally, innovative approaches to reference samples have been described. Chávez-Briones et al. (2023) successfully used long-preserved deciduous teeth as reference material [56], emphasizing the importance of alternative DNA sources in forensic identification.

**Figure 2:**
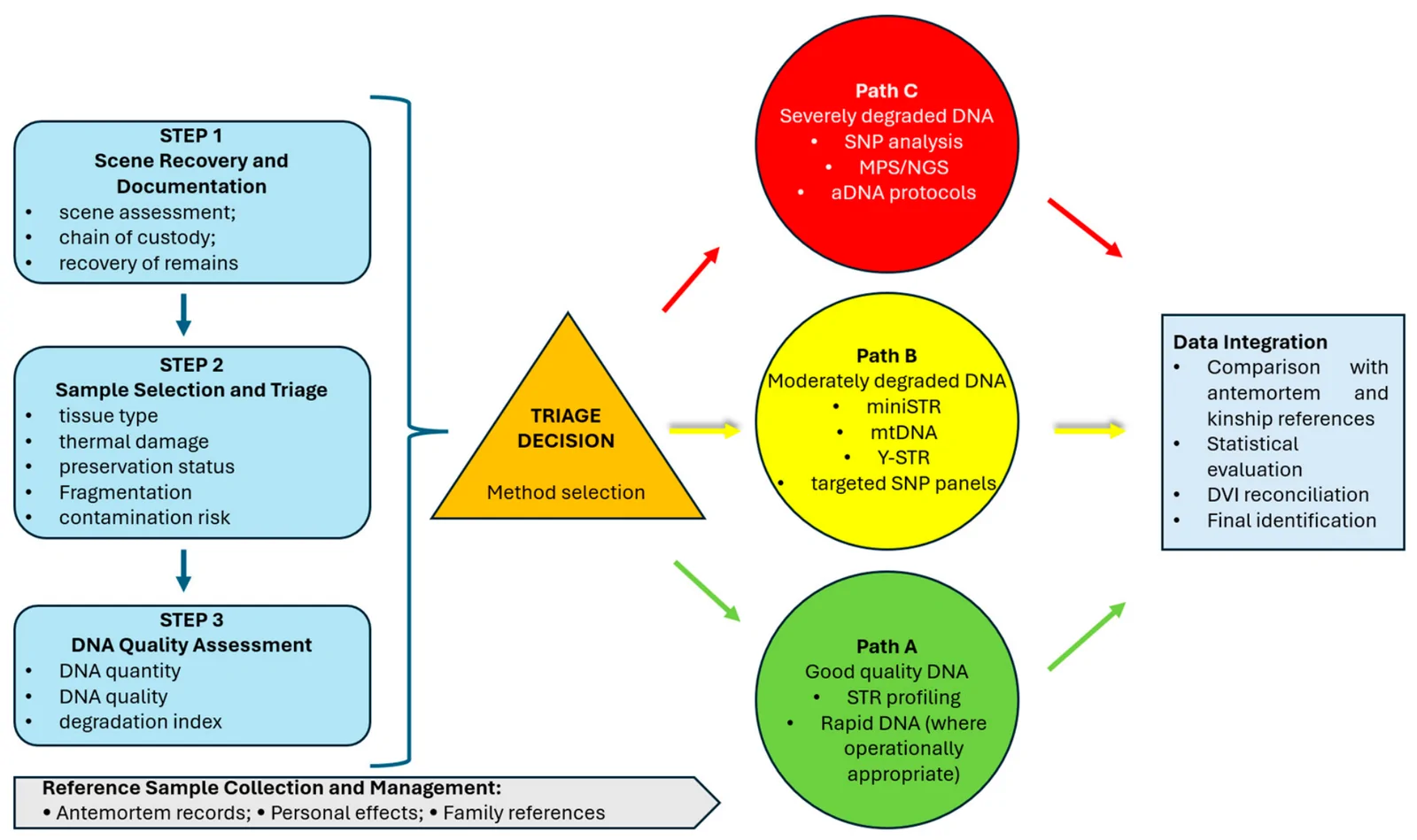
Proposed triage-based workflow for DNA-based identification of burned human remains. Following scene recovery and documentation, biological samples undergo triage according to tissue type, thermal damage, preservation status, fragmentation, contamination risk, DNA quantity, DNA quality, and degradation index. Analytical strategies are subsequently selected based on sample characteristics rather than through a fixed hierarchical sequence. Depending on DNA preservation, STR profiling, mtDNA sequencing, Y-STR analysis, SNP genotyping, rapid DNA platforms, MPS technologies, or ancient DNA workflows may be employed. Postmortem genetic data are then integrated with antemortem and kinship reference samples to support statistical evaluation, DVI reconciliation, and final identification.

The proposed triage-based workflow (Figure 2) synthesizes these elements into an operational framework that can be applied in both routine forensic casework and large-scale DVI.

The proposed workflow incorporates decision nodes based on DNA preservation and sample characteristics. Operationally, this approach can be implemented through a stepwise triage process. Samples showing good preservation and adequate DNA quantity may proceed directly to autosomal STR analysis. Moderately degraded samples may require complementary mtDNA or Y-STR analyses, whereas highly degraded specimens characterized by extensive fragmentation, low DNA quantity, or poor STR performance may be preferentially directed toward SNP-based MPS approaches or ancient DNA methodologies. This decision-making process aims to optimize resource allocation while maximizing identification success.

Based on the findings of this systematic review, several operational recommendations can be proposed to optimize forensic identification of degraded human remains.

From a sampling perspective, a hierarchical approach should be adopted, prioritizing teeth and dense cortical bone due to their superior DNA preservation, followed by protected soft tissues when available. Among dental tissues, molars and other teeth retaining a preserved pulp cavity have repeatedly shown excellent DNA recovery, as the pulp tissue is protected by surrounding enamel and dentin [32,45,53,57]. Likewise, dense cortical bone, particularly from thicker skeletal structures, generally performs better than trabecular bone because of its greater resistance to thermal and environmental degradation [3,42,52,59]. Where vertebral structures are preserved, intervertebral disk fibrocartilage may also be considered as a supplementary DNA source, particularly when bone processing is impractical or time-consuming [68]. Multi-sample collection is strongly recommended to mitigate variability in DNA degradation, as differential thermal exposure may result in substantial differences in DNA preservation even within the same individual [3,52,59,86].

From an analytical standpoint, method selection should be guided by a pre-analytical assessment of sample preservation, DNA quantity, and degradation. Autosomal STR profiling remains highly effective when sufficient nuclear DNA is available; however, highly degraded samples may be more appropriately investigated using mtDNA analysis, SNP-based approaches, ancient DNA methods, or MPS technologies. Therefore, forensic workflows should adopt a flexible triage-based strategy rather than a rigid analytical hierarchy. However, implementation of advanced genomic approaches should also account for laboratory resources, validation status, bioinformatic capabilities, and personnel expertise, ensuring that technology selection remains compatible with local operational capacities.

A structured DVI workflow integrating multidisciplinary expertise and probabilistic kinship analysis is essential to maximize identification success in complex forensic scenarios. An additional critical component is the parallel collection and evaluation of antemortem reference samples and kinship references. Successful identification depends not only on obtaining genetic profiles from postmortem remains but also on the availability and quality of comparative reference data.

These findings collectively support a paradigm shift from a technology-driven approach to a strategy-driven model of forensic identification, in which analytical success is determined by the integration of biological assessment, methodological selection, and operational decision-making.

Future research in forensic genetics is expected to focus on the integration of predictive models, including artificial intelligence-based approaches for estimating DNA preservation and optimizing sample selection [87,88,89,90]. The increasing adoption of MPS technologies will likely expand the analytical capabilities for highly degraded samples.

In addition, the integration of forensic genetics with complementary disciplines such as radiology and anthropology is anticipated to improve identification workflows in complex DVI scenarios. Automation and digitalization of forensic processes, including database management and rapid DNA platforms, will further enhance efficiency and scalability.

Finally, the widespread implementation of advanced genetic technologies raises important ethical and legal considerations, particularly regarding data privacy, consent, and the use of rapid DNA in field settings, which warrant further investigation [91].

### Limitations

Several limitations of the present systematic review must be acknowledged. Importantly, studies were not excluded on the basis of analytical success. Reports describing failed DNA recovery or incomplete genetic profiles were considered eligible when DNA analysis represented a central component of the investigation, thereby reducing the risk of outcome-based selection bias.

First, the included studies are characterized by significant heterogeneity in design, encompassing experimental models, methodological evaluations, and case reports. This variability limits the possibility of direct quantitative comparison and precludes meta-analytical synthesis.

Second, many studies are based on small sample sizes or specific case scenarios, particularly in DVI contexts, which may affect the generalizability of the findings. Additionally, the degree of thermal exposure and environmental conditions is often not standardized across studies, introducing variability in outcomes.

Third, methodological differences in DNA extraction, amplification kits, and analytical platforms may influence results, making it difficult to establish universally applicable thresholds for DNA degradation and recovery.

Fourth, potential publication bias cannot be excluded, as studies reporting successful identification are more likely to be published than those reporting negative results or complete failures. Indeed, although the search strategy was specifically designed to identify studies addressing burned and thermally altered human remains, the selected keywords may not have captured all publications employing alternative terminology, such as “charred remains”, “incinerated remains”, “massively parallel sequencing”, “aviation disasters”, “mass fatality incidents”, or “DVI”. Consequently, some relevant studies may have been missed when these concepts were not explicitly linked to burned human remains in the title, abstract, or keywords. Therefore, certain forensic case reports and DVI investigations may be underrepresented in the present synthesis. In addition, the review did not specifically evaluate economic aspects, cost-effectiveness, or implementation analyses of emerging genomic technologies, which represent an important area for future research.

Finally, although this review focused on DNA-based identification, forensic practice in real-world scenarios is inherently multidisciplinary. Therefore, the integration with other identification methods (anthropology, odontology, radiology) could not be fully explored within the scope of this analysis.

## 5. Conclusions

This systematic review provides an integrated overview of the evolution of forensic genetic identification in burned human remains, spanning traditional STR-based approaches, advanced genomic technologies, and contemporary DVI applications. Beyond summarizing the available evidence, the review identifies recurring determinants of successful DNA recovery, proposes a hierarchical analytical strategy for degraded remains, and translates the findings into an operational workflow applicable to real forensic and humanitarian contexts.

Consistent with the progressive evolution of forensic genetics over recent decades, the studies included in this review demonstrate that DNA degradation should no longer be regarded as an absolute barrier to identification. Advances in ancient DNA methodologies, mitochondrial genome sequencing, targeted SNP recovery, and other MPS-based approaches have progressively extended the analytical limits of forensic genetics in highly degraded and burned remains. Instead, degradation follows predictable patterns that can be strategically addressed through optimized sampling, tailored extraction protocols, and the application of increasingly sensitive analytical techniques. In this context, the traditional reliance on a single method, primarily autosomal STR profiling, has been replaced by a hierarchical and integrative approach, in which multiple genetic systems, including lineage markers and genomic methodologies, are employed in a complementary manner.

Equally important is the recognition that sampling strategy represents a decisive factor in the success of forensic identification. The selection of appropriate biological substrates, such as teeth or dense cortical bone, combined with multi-sample approaches, significantly enhances the likelihood of obtaining usable genetic profiles. At the same time, this review highlights that technological advancements alone are insufficient to guarantee successful outcomes. Rather, the effectiveness of forensic genetics is largely determined by the implementation of structured operational workflows, informed decision-making processes, and multidisciplinary collaboration within DVI frameworks.

Recent developments further indicate a shift toward predictive and time-efficient forensic practices. The adoption of pre-analytical triage methods and the integration of rapid DNA technologies are reshaping traditional workflows, enabling more efficient resource allocation and significantly reducing identification timelines in critical scenarios.

In conclusion, the identification of burned and degraded human remains increasingly benefits from decades of methodological and technological advances that have expanded the recoverability of genetic information from compromised samples. Consequently, successful identification is no longer determined solely by the extent of DNA degradation but by the effective integration of biological assessment, analytical methodology, and operational decision-making within a structured forensic workflow. This paradigm shift firmly establishes DNA-based identification as a cornerstone of modern forensic and humanitarian investigations, with significant implications for future research, standardization, and field application.

## Figures and Tables

**Table 1:** The list of the studied included in the present review.

Authors, Year	Sample Type	Burn/Degradation Level	DNA Method(s)	Main Outcome
Sweet et al., 1995 [32]	Teeth (dental pulp)	Incinerated	STR	Successful identification; teeth protective
Strom et al., 1998 [33]	Blood/charred tissue	Charred	Nested PCR	High sensitivity for degraded DNA
Wickenheiser et al., 1999 [34]	Burned remains	Severe burning	STR	Successful kinship identification
Sivolap et al., 2001 [35]	Bone fragments	Burned + processed	STR	Identification from highly altered remains
von Wurmb-Schwark et al., 2004 [36]	Bone	Cremated (>1000 °C)	STR, mtDNA	DNA unreliable in fully cremated remains
Cerri et al., 2004 [37]	Bone	Burned	mtDNA	mtDNA effective when STR fails
Staiti et al., 2004 [38]	Bone	Degraded/carbonized	STR	Successful STR from degraded tissues
Kapińska et al., 2004 [39]	Bone	Degraded	STR	Phenol–chloroform most effective extraction
von Wurmb-Schwark et al., 2005 [40]	Bone	Burned (modern/historical)	STR, mtDNA	DNA recovery variable over time
Staiti et al., 2008 [41]	Bone	Severely degraded	STR	Identification possible with optimized protocols
Schwark et al., 2011 [42]	Bone	Burn categories	STR, mtDNA	STR success decreases with burn severity
Ricci et al., 2015 [43]	Soft tissue	Burned	STR, Y-STR	Successful DVI
Zgonjanin et al., 2015 [44]	Bone	Burned	STR	Full profiles obtained from skeletal remains
Garriga et al., 2016 [45]	Teeth	100–700 °C	STR	STR success decreases above ~300 °C
Elwick et al., 2018 [46]	Mixed tissues	Degraded	STR kits	Improved sensitivity and inhibitor tolerance
Vullo et al., 2019 [47]	Bone	Degraded	STR	Better DNA yield from preserved samples
Zgonjanin et al., 2019 [48]	Bone	Burned	STR	High profiling success with modern kits
Emery et al., 2020 [49]	Bone/teeth	Burned	STR, aDNA	aDNA improves degraded DNA recovery
Lozano-Peral et al., 2021 [50]	Teeth	Thermal stress	STR	Preferential degradation of long loci
McKinnon et al., 2021 [51]	Bone	Burned	STR	EDTA improves DNA extraction yield
Senst et al., 2021 [52]	Multiple tissues	Altered remains	STR	Tissue selection variable, multi-sampling needed
Varrathyarom et al., 2022 [53]	Bone/teeth	Burned	STR	Teeth yield higher DNA than bone
Emery et al., 2022 [54]	Bone/teeth	Burned	mtDNA (NGS)	Whole mtGenome reconstruction possible
Oh & Park, 2022 [55]	Bone	Cremated	mtDNA	mtDNA viable in extreme degradation
Chávez-Briones et al., 2023 [56]	Bone + teeth	Burned	STR	Identification via deciduous teeth reference
Rahmat et al., 2023 [57]	Teeth	Incinerated	STR, mtDNA	Predictive model for DNA recovery
Snedeker et al., 2023 [58]	Bone	Burned	STR	Optimized extraction improves yield
Macias et al., 2024 [59]	Bone	Burned (color-based)	STR, mtDNA	Color predicts DNA success
Emery et al., 2024 [60]	Bone	Burned	SNP (NGS)	SNP recovery in high degradation
Kugel et al., 2024 [61]	Mixed remains	Mass fatality	DNA analysis	Multidisciplinary DVI approach
Thibodeau-Cadieux et al., 2025 [5]	Bone	100–800 °C	STR	DNA decreases with temperature
Grignani et al., 2025 [62]	Blood	Burned	Methylation	Age estimation possible
Pricop et al., 2025 [63]	Mixed remains	Aviation disaster	DNA analysis	DNA essential in severe burning
Ram et al., 2025 [64]	Mixed tissues	Mass casualty	STR, NGS	~96% identification success
Gianfreda et al., 2025 [65]	Soft tissue	Carbonized	STR	Full profiles via targeted sampling
Weinthal et al., 2025 [66]	Mixed remains	Extreme DVI	Rapid DNA	Identification within hours
Sarwar et al., 2026 [67]	Bone/tissue	Aircraft crash	STR, mtDNA	~97% identification success

## Data Availability

No new data are used in this review.

## Supplementary Materials

The following supporting information can be downloaded at: https://www.mdpi.com/article/10.3390/genes17080881/s1, Table S1: Qualitative Quality Assessment of Included Studies.

## Abbreviations

The following abbreviations are used in this manuscript:

CE	capillary electrophoresis
MPS	massively parallel sequencing
STRs	short tandem repeats
DVI	disaster victim identification
NGS	next-generation sequencing
mtDNA	mitochondrial DNA
PRISMA	Preferred Reporting Items for Systematic Reviews and Meta-Analyses
SNP	single-nucleotide polymorphism
PCR	polymerase chain reaction
aDNA	ancient DNA
FDP	forensic DNA phenotyping
