# Supplementary material for: Genetic Identification of Burned Human Remains: A Systematic Review
Source: Genes (Basel). 2026 Jul 28;17(8):881. doi: 10.3390/genes17080881 (PMC13512412; doi:10.3390/genes17080881)
Supplement: Supplementary file 1 [file genes-17-00881-s001.zip › supplementary table 1.pdf]

Supplementary Table S1. Qualitative Quality Assessment of Included Studies

**Study**	**DNA robustness**	**Sample size**	**Degradation control**	**Reproducibility**	**DVI applicability**
Sweet 1995	3	2	4	4	4
Strom 1998	4	2	4	4	3
Wickenheiser 1999	4	2	2	3	5
Sivolap 2001	4	2	3	3	5
von Wurmb-Schwark 2004	3	3	5	4	3
Cerri 2004	4	2	3	3	4
Staiti 2004	4	3	4	4	3
Kapińska 2004	3	3	4	4	2
von Wurmb-Schwark 2005	3	3	4	4	3
Staiti 2008	4	2	4	4	3
Schwark 2011	4	3	5	5	4
Ricci 2015	4	3	3	3	5
Zgonjanin 2015	4	2	3	3	5
Garriga 2016	4	3	5	5	3
Elwick 2018	5	3	4	5	3
Vullo 2019	4	4	4	5	4
Zgonjanin 2019	4	3	3	3	4
Emery 2020	5	3	4	5	4
Lozano-Peral 2021	4	3	5	5	3
McKinnon 2021	4	3	5	5	3
Senst 2021	4	5	3	3	5
Varrathyarom 2022	4	3	4	4	4
Emery 2022	5	3	4	5	4
Oh & Park 2022	4	2	4	4	4
Chávez-Briones 2023	4	2	3	3	5
Rahmat 2023	5	3	5	5	4
Snedeker 2023	5	3	4	5	4
Macias 2024	5	3	5	5	4
Emery 2024	5	3	4	5	4
Kugel 2024	3	5	2	2	5
Thibodeau-Cadieux 2025	4	3	5	5	3
Grignani 2025	5	3	3	4	4
Pricop 2025	3	4	2	2	5
Ram 2025	5	5	3	3	5
Gianfreda 2025	5	3	3	3	5
Weinthal 2025	5	4	3	4	5
Sarwar 2026	5	5	3	4	5
